# Monitoring of aphid flight activities in seed potato crops in Serbia

**DOI:** 10.3897/zookeys.319.4315

**Published:** 2013-07-30

**Authors:** Andja Vučetić, Tanja Vukov, Ivana Jovičić, Olivera Petrović-Obradović

**Affiliations:** 1 University of Belgrade, Faculty of Agriculture, Nemanjina 6, Zemun, Belgrade, Serbia; 2 University of Belgrade, Institute for Biological Research “Siniša Stanković”, Belgrade, Serbia

**Keywords:** Aphids, potato, Shannon-Weaver index diversity, Morisita-Horn similarity index, vectors of viruses

## Abstract

Aphid flight activities in seed potato fields have been studied by the yellow water traps. It is a good method for monitoring aphids as vectors of viruses, but this study also showed it is a suitable method for insect-diversity research. During the four-year studies, over 11.500 specimens were collected and a total of 107 different taxa of aphids were identified. The most abundant species were polyphagous species, such as: *Acyrthosiphon pisum* (Haris), *Aphis fabae* Scopoli, *Aphis gossypii* Gloverand *Brachycaudus helichrysi* (Kaltenbach). The results of the studies show that diversity of aphids in different regions of Serbia is similar regardless of the altitude and the diversity of terrain. At most sites it ranged from 2 to 3. The highest value was recorded in Begeč, locality in northern part of Serbia, in year 2008, and it was 2.92. The maximum values of the Shannon-Weaver diversity index at all sites were recorded in the first weeks of the monitoring of aphid flight activities. Morisita-Horn similarity index shows no significant differences between sites regardless of altitudes. The sites are grouped by year, not by similarity of relief. In spite of these results, the Chi-square analysis showed highly significant difference in vector frequencies among seasons and sites with more pronounced differences for PVY. As a consequence of differences in vector frequencies, the vector pressure index in some regions was different also. The number of vectors and vector pressure index vary depending on the altitude of localities. At localities at altitudes under 1000 m, they were high. The highest index was at Kotraža, locality in central part of Serbia, in 2007, when PVY index exceeded the value of 180, while for PLRV it was 60. At high altitudes on mountain Golija, above 1100 m, the number of aphids was low, as well as the vector pressure index which indicates that these regions are suitable for producing virus-free seed potato.

## Introduction

Aphids (Aphididae, Hemiptera) are the most efficient vectors of plant pathogenic viruses therefore they cause serious problems in potato growing. Production of healthy seed potatoes is possible in conditions of reduced number of aphids and their ability to come into contact with the plant and transfer the virus (Robert and Bourdin 2001). Two most important potato viruses are Potato Virus Y (PVY) and Potato Leafroll Virus (PLRV). Seed quality depends directly on the infection level (Salazar 1996).

After infection of leaves, the virus is translocated into the tubers. In some countries, earlier sowing and haulm destruction is carried out at critical period of virus infection (Van Harten 1983). It is a good way to stop virus transmission from leaves to tubers. In Serbia the maximum aphid flight activities and at the same time the maximum vector activities occur end of May-early June (Petrović-Obradović 2003). In that period, potato is at early stages of growth and desiccation is not possible. That can completely interrupt plant’s vegetation and the yield would be lost. Because of similar vegetation complexity, relief, climate and aphid fauna, situation is similar in neighboring countries in southeastern Europe. In this region, it is necessary to find some other way for the production of healthy seed potato. One possibility is to find localities with a lower number of aphids and inoculum sources. The success of agricultural production depends on the biodiversity of an area, the number of present organisms, which may have a positive or a negative impact (Dueli 1997, Laznik et. al. 2010). Diversity of aphids is just a segment of biodiversity of an area, but significant in the aspect of ecology and crop production, in this case production of seed potatoes.

The aim of these studies was to determine the biodiversity of aphids and similarity in aphid composition between different regions of Serbia. Also, the aim of these studies was to determine differences in vectors frequency among different sites in Serbia and to calculate the pressure of vectors for the two most important potato viruses (Potato Virus Y – PVY and Potato Leafroll Virus – PLRV), and thus determine which areas are suitable for the cultivation of healthy seed potatoes.

## Material and methods

**Figure 1.**
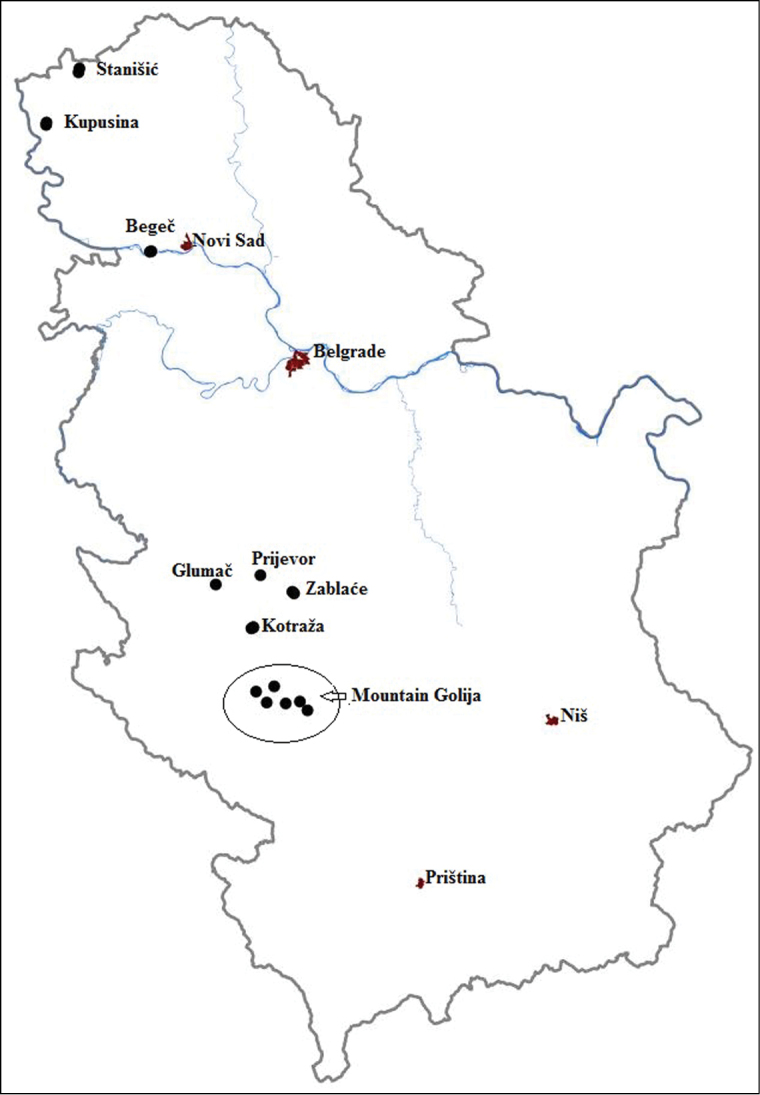
Map of Serbia with monitored aphid flight activities sites. Coordinates of localities: Begeč 2007 (45°13'26"N, 19°36'53"E), Begeč 2008 (45°13'34"N, 19°37'23"E), Glumač 2008 (43°52'27"N, 20°1'1"E), Golija 1 2007 (43°27'45"N, 20°20'58"E), Golija 2 2007 (43°21'58"N, 20°32'12"E), Golija 1 2008 (43°26'25"N, 20°14'56"E), Golija 2 2008 (43°23'36"N, 20°24'53"E), Golija 1 2009 (43°24'6"N, 20°29'38"E), Golija 2 2009 (43°23'48"N, 20°18'31"E), Kotraža 2007 (43°42'7"N, 20°13'49"E), Kotraža 2008 (43°42'17"N, 20°13'34"E), Kotraža 2010 (43°41'49"N, 20°13'9"E), Kupusina 2008 (45°44'24"N, 19°0'6"E), Kupusina 2009 (45°43'49"N, 18°59'58"E), Stanišić 2008 (45°56'48"N, 19°10'51"E), Stanišić 2009 (45°57'7"N, 19°9'10"E), Stanišić 2010 (45°57'46"N, 19°11'6"E), Prijevor (43°54'49"N, 20°16'2"E), Zablaće 2 2009 (43°50'51"N, 20°26'51"E), Zablaće 1 2010 (43°50'27"N, 20°27'25"E).

Aphid flight activity was studied in different areas of Serbia in twenty sites for four years (2007–2010). These 20 sites belong to the three major potato growing areas in Serbia. The first area is in northern part of Serbia under the altitudes of 80 m (localities: Begeč, Stanišić, Kupusina). The second one is in central part at altitudes of 400 – 900 m (localities: Kotraža, Zablaće, Prijevor, Glumač), and the third one is in southern part at altitudes above 1100 m (localities on mountain Golija) (Fig. 1). Monitoring of aphid flight activity was conducted by using yellow water traps. Yellow water traps were placed in potato crops (4traps/1ha) immediately after the emergence of potato. Traps have been raised gradually to be visible for aphids during the growth of the crop. Samples were taken once per week until drying of the above-ground mass. Aphids were identified using a stereoscopic microscope (Bio-optica, Italy, Type: 1000) and keys for identification of alatae aphids (Taylor 1984, Jacky and Bouchery 1988, Remaudiere and Seco Fernandez 1990).

Shannon–Weaver index was used for the analysis of biodiversity (Krebs 1989). A diversity index is a quantitative measure that reflects how many different species there are in a dataset, and simultaneously takes into account how evenly individuals are distributed among those types. It is always ranging between 0 (indicating low community complexity) and 4 (indicating high community complexity). Morisita–Horn similarity index was used to calculate the similarities in aphid composition among the sites (Magurran 2004). This index takes into account composition and richness of fauna and successfully compares samples of different size. The maximum value of this index is 1. The cluster analysis on the basis of Morisita–Horn index after the UPGA method was conducted. Comparisons of number of aphids that are vectors for PVY and PLRV viruses among localities were performed with Chi-square analysis. Similarity Percentage analysis (SIMPER) (Clarke 1993) was applied for determination of the contribution of each aphid species, which are identified as virus vectors, to the similarity (the dissimilarity) between all localities and years of collections. The Bray-Curtis measure was calculated after logarithmic transformation of the data. The risk of infection of potato by viruses was shown as a cumulative index vector pressure which was calculated using the Relative Transmission Efficiency value of aphids, known vectors of potato viruses (http://aphmon.csl.gov.uk/info.cfm).

## Results

During the four-year studies, over 11.500 specimens were collected and a total of 107 different taxa of aphids were identified. Seventy five different taxa were identified to the species level, thirty two to genus level. Thirty six heteroecious species and thirty nine monoecious species were identified (Table 1). According to the data Petrović-Obradović (2003), 60 aphid species, which is about 17% of identified species in Serbia, are heteroecious. During these researches, almost 50% of identified species were heteroecious, which are commonly polyphagous species and some of them are the most important vectors of potato viruses. Two invasive species were found during this research, *Trichosiphonaphis polygonifoliae* (Shinji), which had been recently discovered on its host plant (Petrović-Obradović et al. 2010) and *Macrosiphum albifrons* Essig which had been found for the first time in Serbia during this research.

**Table 1.** Identified aphid taxa (^PVY^ vectors of PVY, ^PLRV^ vectors of PLRV).

**Monoecious species**	**Heteroecious species**	**Genus**
*Acyrthosiphon cyparissiae* (Koch)	*Anoecia corni* (F.)	*Acyrthosiphon* spp.
*Acyrthosiphon malvae* (Mosley)	*Aphis fabae* Scopoli^PVY /PLRV^	*Amphorophora* spp.
*Acyrthosiphon pisum* (Haris)^PVY^	*Aphis gossipii* Glover^PLRV^	*Anoecia* spp.
*Amphorophora rubi* (Kaltenbach)	*Aphis nasturtii* Kaltenbach	*Aphis* spp.
*Aphis craccivora* Koch	*Aphis spiraecola* Patch	*Brachycaudus* spp.
*Aphis idaei* van der Goot	*Aphis sambuci* L.	*Capitophorus* spp.
*Aphis pomi* De Geer	*Aulacorthum solani* (Kaltenbach)^PVY/PLRV^	*Cavariella* spp.
*Atheroides serrulatus* Haliday	*Brachycaudus cardui* (L.)	*Chaitophorus* spp.
*Brevycorinae brassicae* (L.)	*Brachycaudus helichrysi* (Kaltenbach)^PVY^	*Cinara* spp.
*Callipterinela calliptera* (Hartig)	*Capitophorus eleagni* (del Guercio)	*Dysaphis* spp.
*Callipterinela tuberculata* (von Heyden)	*Cavariella theobaldi* (Gillete and Bragg)	*Eriosoma* spp.
*Chaitophorus populialbe* (Boyer de Fonscolombe)	*Cryptomyzus galeopsidis* (Kaltenbach)	*Euceraphis s* pp.
*Cinara tujafilina* (del Guercio)	*Cryptomyzus ribis* (L.)	*Forda* spp.
*Ctenocallis setosus* (Kaltenbach)	*Dysaphis plantaginea* (Passerini)	*Hyadaphis* spp.
*Drepanosiphum aceris* Koch	*Eriosoma ulmi* (L.)	*Hyperomyzus* spp.
*Eucalipterus tiliae* (L.)	*Forda marginata* Koch	*Macrosiphoniella* spp.
*Euceraphis betulae* (Koch)	*Hyadaphis foeniculi* (Passerini)	*Microlophium* spp.
*Eriosoma lanigerum* (Hausmann)	*Hyalopterus pruni* complex	*Myzocallis* spp.
*Hyadaphis polonica* Szelegiewicz	*Hyperomyzus lactuce* (L.)	*Myzus* spp.
*Lachnus roborus* (L.)	*Hyperomyzus pallidus* Hille Ris Lambers	*Ovatus* spp.
*Lipaphis erysimi* (Kaltenbach)	*Hyperomyzus picridis* (Börner and Blunck)	*Pemphigus* spp.
*Macrosiphum albifrons* Essig	*Macrosiphum euphorbiae* (Thomas)^PVY/PLRV^	*Periphyllus* spp.
*Macrosiphum funestrum* (Macchiati)	*Macrosiphum rosae* (L.)	*Protaphis* spp.
*Megoura viciae* Buckton	*Metopolophium dirhodum* (Walker)^PVY^	*Protrama* spp.
*Megourella purpurea* Hille Ris Lambers	*Myzus cerasi* (Fabricus)	*Rhopalosiphum* spp.
*Myzocallis castanicola* Baker	*Myzus persicae* (Sulzer)^PVY/PLRV^	*Semiaphis* spp.
*Myzocallis occidentalis* Remaudie et Nieto Nafria	*Nasonovia ribis-nigri* (Mosley)	*Sipha* spp.
*Myzodium modestum* (Hottes)	*Phorodon humuli* (Schrank)^PLRV^	*Subsaltusaphis* spp.
*Myzus ligustri* (Mosley)	*Rhopalomyzus poae* (Gill)	*Tetraneura* spp.
*Ovatus inulae* (Walker)	*Rhopalosiphoninus staphylleae* (Koch)^PLRV^	*Therioaphis* spp.
*Phyllaphis fagi* (L.)	*Rhopalosiphum maidis* (Fitch)	*Tuberculatus* spp.
*Pterocallis alni* (de Geer)	*Rhopalosiphum nimfaeae* (L.)	*Uroleucon* spp.
*Schizaphis graminum* (Rondani)	*Rhopalosiphum padi* (L.)^PVY^	
*Sipha elegans* del Guercio	*Sitobion fragariae* (Walker)	
*Sipha maydis* Passerini	*Smynthurodes betae* Westwood	
*Sitobion avenae* (Fabricius)	*Trichosiphonaphis polygonifoliae* (Shinji)	
*Therioaphis trifolii* (Monell)		
*Tinocallis platani* (Kaltenbach)		
*Wahlgreniella ossiannilssoni* Hille Ris Lambers		

**Figure 2.**
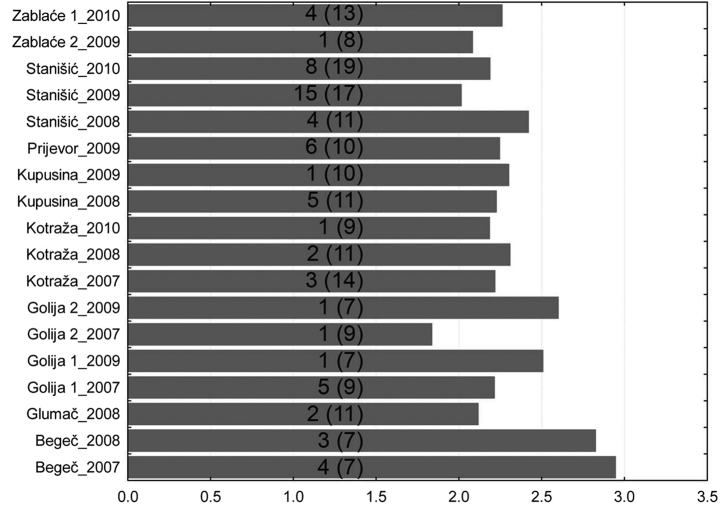
Maximum of Shannon–Weaver index per locality (number in brackets - number of weeks of monitoring aphid flight activities, number without brackets - week with maximum value of Shannon–Weaver index).

Results from 20 different localities were used for the analysis of biodiversity using Shannon–Weaver index. The maximum values of the Shannon–Weaver index diversity at all sites were recorded in the first weeks of the monitoring of aphid flight activities (Fig. 2).

**Figure 3.**
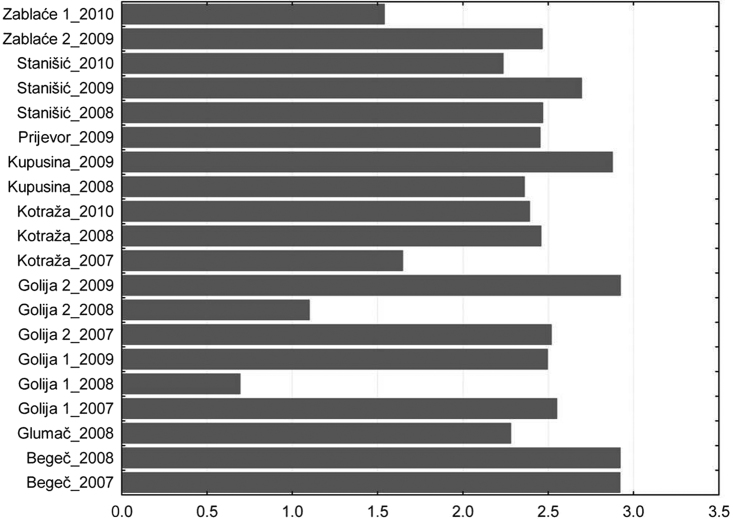
Total Shannon–Weaver index diversity per locality.

The results of studies show that diversity of aphids in different regions of Serbia is similar regardless of the altitude and the diversity of terrain. At most sites, it ranged from 2 to 3. The highest value was recorded in Begeč in 2008, where it was 2.92. The lowest values were on the mountain Golija in 2008, where on the locality at the lower altitudes was 0.69 and at the higher altitudes was 1.098. In that year, in two localities on this mountain, a total of 5 aphid specimens were caught (Fig. 3).

**Figure 4.**
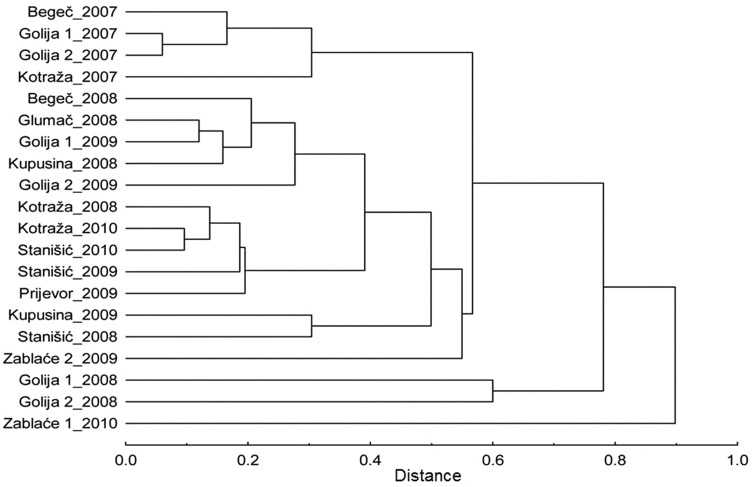
Dendrogram shows similarity between the sites, constructed on the basis of Morisita–Horn similarity index.

Also, Morisita–Horn similarity index shows no significant differences in aphid composition between sites regardless of altitudes. The cluster analysis on the basis of this index was carried out (Fig. 4). The sites are grouped by year, not by similarity of relief. Sites on the mountain Golija were clearly separated from the rest because of the low number of aphids in the year 2008. In the locality Zablaće, five aphid species were caught which were not recorded previously in other localities during these studies. These species are: *Chaitophorus populialbae* (Boyer de Fonscolombe), *Myzocallis castanicola* Baker, *Myzocallis occidentalis* Remaudie et Nieto Nafria, *Protrama* spp. and *Sminthuroides betae* Westwood.

Important potato virus vectors such as: *Acyrthosiphon pisum* (Haris), *Aphis fabae* Scopoli, *Aphis gossypii* Glover, *Aulacorthum solani* (Kaltenbach), *Brachycaudus helichrysi* (Kaltenbach), *Macrosiphum euphorbiae* (Thomas), *Metapolophium dirhodum* (Walker), *Myzus persicae* (Sulzer) and *Rhopalosiphum padi* (L.) were found in almost all localities, but in different numbers. The most important vector of potato viruses *Myzus persicae*, was found in all localities, but in high number just in localities Begeč and Kotraža in 2007.

The Chi–square analysis showed highly significant difference in vector frequencies among seasons and sites, with more pronounced differences for PVY (Table 2).

**Table 2.** Results from Chi–square analysis used to compare the different sites by the number of vectors for PVY and PLRV viruses.

PVPLRV	Begec 2007	Begec 2008	Glumac 2008	Golija 1 2007	Golija 1 2009	Golija 2 2007	Golija 2 2009	Kotraza 2007	Kotraza 2008	Kotraza 2010	Kupusina 2008	Kupusina 2009	Prijevor 2009	Stanisic 2008	Stanisic 2009	Stanisic 2010	Zablace 1 2010	Zablace 2 2009
Begec 2007		***	***	***	***	***	***	***	***	***	***	***	***	***	***	***	***	***
Begec 2008	***		ns	***	***	***	***	***	***	***	***	***	***	***	***	***	***	***
Glumac 2008	***	*		***	***	***	***	***	***	***	***	***	***	***	***	***	***	***
Golija 1 2007	***	***	***		***	ns	ns	***	**	ns	***	ns	*	ns	*	ns	ns	**
Golija 1 2009	***	***	***	***		***	***	***	***	***	ns	***	***	***	***	***	***	***
Golija 2 2007	***	***	***	ns	***		ns	***	*	ns	*	ns	ns	ns	ns	ns	ns	*
Golija 2 2009	***	***	***	***	***	***		***	***	ns	**	ns	*	ns	*	ns	ns	**
Kotraza 2007	***	***	***	***	***	***	***		***	***	***	***	***	***	***	***	***	***
Kotraza 2008	***	***	***	***	***	***	*	***		*	***	***	*	***	**	*	***	**
Kotraza 2010	***	***	***	*	***	***	ns	***	**		***	***	ns	**	ns	ns	**	*
Kupusina 2008	***	***	***	***	**	***	***	***	**	***		***	***	***	***	***	ns	***
Kupusina 2009	***	***	***	***	***	***	ns	***	***	***	***		**	ns	ns	ns	*	***
Prijevor 2009	***	***	***	ns	***	ns	ns	***	***	ns	***	***		***	ns	ns	***	***
Stanisic 2008	***	***	***	**	***	*	***	***	***	**	***	**	**		ns	*	ns	*
Stanisic 2009	***	***	***	***	***	***	**	***	***	*	***	***	***	***		***	***	***
Stanisic 2010	***	***	***	*	***	**	ns	***	***	ns	***	***	ns	***	***		ns	*
Zablace 1 2010	***	***	***	***	***	***	*	***	*	ns	*	**	ns	***	***	ns		ns
Zablace 2 2009	***	***	***	***	***	***	**	***	***	*	***	***	***	***	***	**	***	

* - significant differences at a level of significance α = 0.05

** - significant differences at a level of significance α = 0.01

*** - significant differences at a level of significance α = 0.001

ns - not significant differences

Similarity Percentage analysis shown that similarities in presence of PVY vectors between localities in 2007 year was almost 60%. In that year the most common species was *Brachycaudus helichrysi*, which was dominant species in all localities, but in localities Begeč and Kotraža it was found in large number. In the locality Kotraža over 1500 specimens were caught during the monitoring period. In the next years, large number of the specimens of this species has not been repeated, and because of that there was low similarity percent between localities in this year and localities in the fallowing years. In 2008 and 2010 similarity percent between localities were 70%, but in 2009 it was 50%. In all those years the most common vector species were *Aphis fabae* and *Acyrthosiphon pisum* which were most responsible for high percent of similarities between localities. In all years the least number of aphids was recorded in localities in mounting Golija at altitudes above 1100m. There were recorded no significant differences between these localities and locality Prijevor because of low number of aphids in this locality and similar number of species *Aphis fabae* and *Aulacorthum solani*. Similarity between localities in presence of PLRV vectors was around 70% in 2008 and 2010. In 2007 it was 51% and in 2009 just 44%. In all years the most common vector species was *Aphis fabae*. The best average dissimilarity was recorded between localities Begeč in 2007 and 2008 and all others localities because of constantly high number of vectors in this area. Especially high percent was recorded between this locality and localities Golija 2 in all three years, and it was 60%.

**Figure 5.**
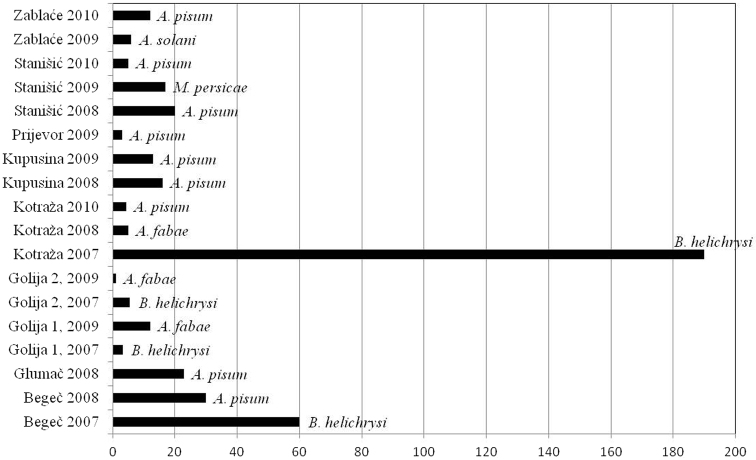
Vector pressure index for PVY and most important vector per locality.

**Figure 6.**
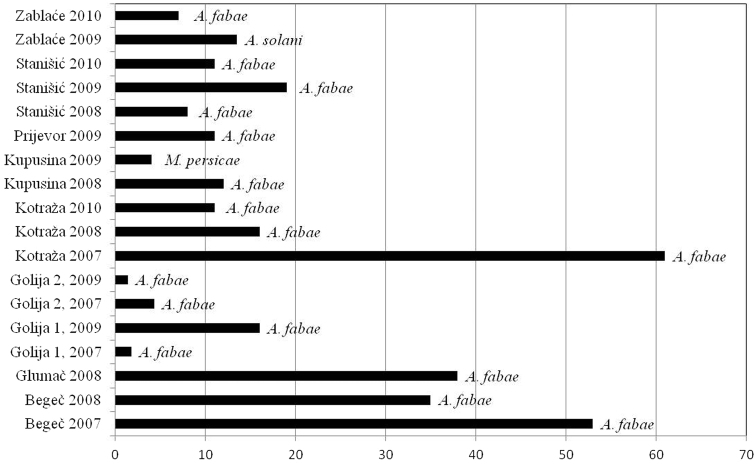
Vector pressure index for PLRV and most important vector per locality.

As a consequence of differences in diversity among sites and difference in vector frequencies, the vector pressure index in some regions was different also. The lowest vector number and the lowest vector pressure were observed in Golija mountain area during the study. In localities at above 1100m in Golija, vector pressure index newer exceeded 10. The highest value was recorded in locality Kotraža in 2007 (at altitudes of 850m), when pressure of vectors for PVY exceeded 180, and for PLRV exceeded 60. In the following years, these high values were not repeated. Pressure of vectors for both viruses was constantly high in locality Begeč. In other localities, at the lowest altitudes, Kupusina and Stanišić, values of pressure vectors were low, but reached their maximums early in the season (Figs 5, 6).

## Discussion

In the studies of biodiversity, in order to obtain a result with a scientific value, it is necessary to standardize sampling method, i.e. adapt it to target organisms. Different types of traps are the best known and the most widely used methods of sampling (Duelli 1997). Yellow water traps are often used for monitoring of aphid flight activities in potato fields (Sigvald 1989, Milošević and Petrović 1997, Kuroli and Lantos 2006, Kotzampigikis et al. 2008), but had not been used before for the studies of biodiversity of aphids. During these studies, they proved to be a good method, giving valid results. Shannon-Weaver diversity index is not an absolute indicator of the diversity of a certain area, it is used to compare different localities or the same locality in different years or weeks of a year. For this purpose it was used in these researches.

The values of Shannon–Weaver diversity index varied during the growing season. Temperature changes and rain influence the abundance of aphids (Morgan 2000), which also results in changes in the values of this index. However, in most localities the highest values were recorded in the first half of the monitored period, i.e. in spring. The total values of the index were similar among different sites, regardless of altitude. Due to the fact that Shannon–Weaver index takes into account the number of species and frequency of each species’ individuals, localities with large differences in the number of aphids had similar index values. The highest values were recorded in locality Begeč, at an altitude of 80 m. Morisita–Horn index takes into account the diversity of species and number of individuals, and it showed that there are not large differences between different localities, i.e. different localities have similar richness of the species. Locality Zablaće was clearly separated from other localities because of the five species which were recorded only in this locality during investigations.

In spite of similar values of Shannon–Weaver diversity index among different localities, participation of the vectors in it is different. The Chi–square analysis showed highly significant difference in vector frequencies among seasons and sites, with more pronounced differences for PVY. As a consequence of differences in vector frequencies, the vector pressure index in some regions was different also. In areas at lower altitudes such as Begeč, a higher number of vector species was registered, as well as more individuals of each present species. *Myzus persicae*, the most important vector of viruses was found in most localities, but in high number only in localities Begeč and Kotraža, while at the localities above 1100 m it was registered very rarely and in a low number. In localities under 900m, potato sowing is usually done in April, while there is an intensive growth of potato in May when aphid flight is at maximum and virus infection risk is the highest. Potato is the most sensitive in the first development phases, until flowering (DiFonso et al. 1994). Production of virus free seed potatoes is possible if the pressure of vectors does not exceed the value of 10–15 by the end of June – early July (Basky 2002). In localities above 1000m, potato sowing is usually done at the end of May or beginning of Jun, depending on the ambient temperature. Except a lower number of aphids at higher altitudes, later sowing leads to avoiding the periods of aphid maximum flight and the risk of virus infection is reduced. Also, at higher altitudes, agricultural production is not intensive and the possibilities for isolated production are stronger.

Results of these studies show that only in localities at high altitudes, such as mountain Golija, it is possible to grow healthy, virus free seed potato. This research indicated that the potential of other mountainous regions of Serbia is also high and that Serbia has the capacity for production of quality seed potato. Also, this research may have relevance and application in other neighboring countries, too, because of similar relief, vegetation composition, composition of the fauna of aphids, and the possibility of crop production.
